# The Case for Open Preprints in Biology

**DOI:** 10.1371/journal.pbio.1001563

**Published:** 2013-05-14

**Authors:** Philippe Desjardins-Proulx, Ethan P. White, Joel J. Adamson, Karthik Ram, Timothée Poisot, Dominique Gravel

**Affiliations:** 1 Theoretical Ecosystem Ecology laboratory, Université du Québec à Rimouski, Rimouski, Quebec, Canada; 2 Quebec Center for Biodiversity Science, McGill University, Montreal, Quebec, Canada; 3 Department of Biology, Utah State University, Logan, Utah, United States of America; 4 Ecology, Evolution and Organismic Biology, University of North Carolina at Chapel Hill, Chapel Hill, North Carolina, United States of America; 5 Environmental Science, Policy, and Management, University of California, Berkeley, California, United States of America; 6 International Network for Next-Generation Ecology

## Abstract

Biologists should submit their preprints to open servers, a practice common in mathematics and physics, to open and accelerate the scientific process.

## Introduction

Public preprint servers allow authors to make manuscripts publicly available before, or in parallel to, submitting them to journals for traditional peer review. The rationale for preprint servers is fundamentally simple: to make the results of research available to the scientific community as soon as possible, instead of waiting until the peer-review process is fully completed. Sharing manuscripts using preprint servers has numerous advantages, including: 1) rapid dissemination of work-in-progress to a wider audience; 2) immediate visibility of the research output for early-career scientists; 3) improved peer review by encouraging feedback from the entire research community; and 4) a fair and straightforward way to establish precedence.

Open preprint servers offer a great opportunity for open science, especially if the community embraces the idea of discussing preprints. Initiatives like Haldane's Sieve (http://haldanessieve.org/), a new blog discussing arXiv papers in population genetics, can help make arXiv attractive for scientists looking to promote their work [1]. These initiatives are important to fully exploit the potential of open preprint servers. Posting preprints online increases the community of available informal peer reviewers, and uses the internet for its original community-building purposes.

Preprints began to gain popularity 20 years ago with the advent of arXiv, an open preprint server widely used in physics and mathematics [2]. Preprints are also integral to the culture of other scientific fields. Paul Krugman noted that, in economics, the “traditional model of submit, get refereed, publish, and then people will read your work broke down a long time ago. In fact, it had more or less fallen apart by the early 80 s” [3]. In addition to a section on arXiv, economists have the RePEc (Research Papers in Economics) initiative, which aims to create an archive of working papers, manuscripts, and book chapters.

Despite the success of this approach in other fields, most manuscripts in biology are not posted to preprint servers and are therefore not seen by more than a handful of other scientists prior to publication. In this article, we highlight the advantages of open preprint servers for both scientists and publishers, discuss the preprint policies of major publishers in biology, and describe the main options to publish preprints (Box 1, Table 1).

**Table 1:** Popular options for preprints.

Website	Free	Comments	Private	Peer-Reviewed	DOI	Version-Control	Other Content
arXiv.org	Yes	No	No	No	No	No	No
figshare.com	Yes	Yes	Yes	No	Yes	No	Yes
peerj.com	1/yr	Yes	Yes	No	Yes	No	No
f1000research.com	No	Yes	No	Yes	Yes	No	No
github.com	Yes	Yes	Yes	No	No	Yes	Yes

**Free:** Can preprints be submitted for free. **Comments:** Support for online comments. **Private:** Support for private preprints. **Peer-Reviewed:** Whether the preprints are peer-reviewed on the server. **DOI:** Each item is assigned a unique digital object identifier. **Version-Control:** Is the preprint stored using a version-control system with the complete history of modifications? **Other content:** Can upload figures, videos, datasets, code.

**Box 1. Preprint Server Roundup**

**arXiv**

arXiv (http://arxiv.org/) is the most widely used preprint server today, and its use is almost universal in some branches of mathematics and physics. arXiv has a system of moderators and endorsers. At least one author of a paper must be an endorser that has either previously submitted a paper or has received permission to submit. Moderators have the power to change the classification of a manuscript.

**figshare**

figshare (http://figshare.com) is an open server allowing scientists to submit any research output: manuscript, figures, datasets, videos, theses, presentations, and so on. There are no rules to limit what constitutes a research output and, unlike arXiv, there is no endorser system. A flexible tag system is used to classify each item.

**PeerJ**

PeerJ (https://peerj.com/) is a new commercial open access publisher focused on the biological sciences that provides a preprint server and a peer-reviewed journal. Preprints can optionally be made private. One preprint per year can be posted for free, with a onetime (i.e., lifetime) fee for unlimited public preprints. Preprints can be posted to PeerJ regardless of where they will be submitted for publication.

**F1000Research**

Whereas arXiv, figshare, and PeerJ offer an option to submit a manuscript without having it reviewed, papers submitted to F1000Research (http://f1000research.com/) will eventually be reviewed. Thus, F1000Research offers a hybrid model with publicly available manuscripts at time of submission and standard peer reviews that occur as part of the submission process. Manuscripts are considered “accepted” and will only be indexed after two positive referee responses.

**GitHub**

This manuscript was developed entirely as an open project on GitHub (https://github.com/). GitHub is one of several hosting services for collaborative development using the Git version control system (VCS). It allows numerous contributers to work asynchronously on the same project, often in parallel branches, all of which can be effortlessly merged and version controlled. Git is primarily used for software development [13], but it provides a powerful tool to collaborate on every step of the manuscript development process [14].

**Other options**

Scientific publishing is more diversified than ever. There are now many alternative options for submitting articles before formal publication. For example, social networks such as ResearchGate (http://www.researchgate.net/) can be used to submit preprints [15]. Also, if GitHub pushes openness further by opening the writing process, open notebooks go even further by opening the entire scientific process [16].

## The Case for Public Preprints

**Figure 1:**
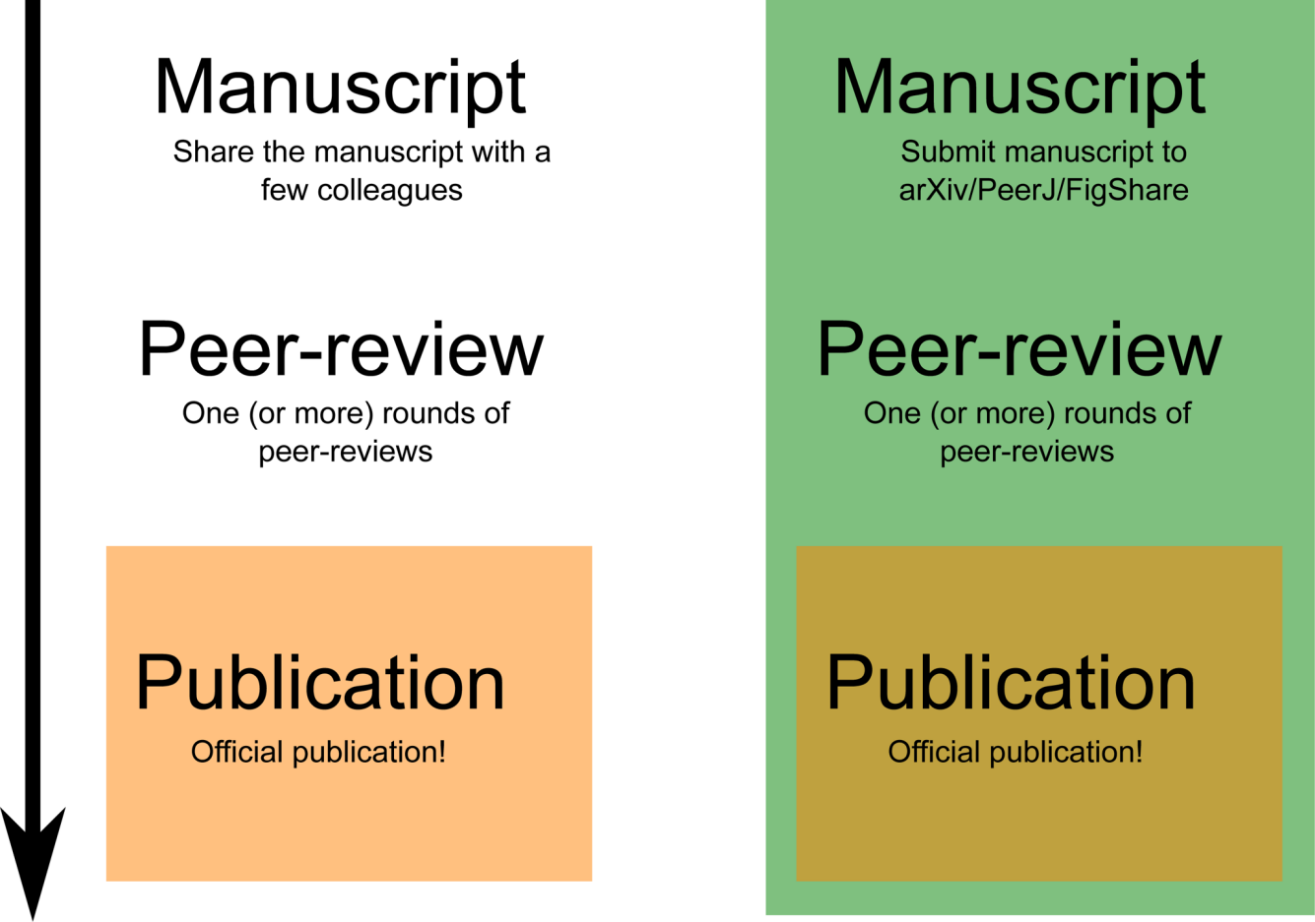
It can take several months before a submitted paper is officially published and citable. Meanwhile, few people are aware of the research that has been done since, typically, only close colleagues are given access to the preprints. With public preprint servers, the science is immediately available and can be openly discussed, analyzed, and integrated into current research.

The first and most often discussed advantage of open preprints is speed (Figure 1). The time between submission and the official publication of a manuscript can be measured in months, sometimes in years. For all this time, the research is known only to a select few: colleagues, editors, and reviewers. Thus, the science cannot be used, discussed, or reviewed by the wider scientific community. In a recent blog post, C. Titus Brown noted how posting a paper on arXiv quickly led to a citation (arXiv papers can be cited), and his research was used by another researcher [4]. The current system of hiding manuscripts before acceptance poses problems for both scientists and publishers. Manuscripts that are unknown cannot be used and thus take more time to be cited. It has been shown that high-energy physics, with its high arXiv submission rate, has the highest immediacy among physics and mathematics [5]. Immediacy measures how quickly articles are cited.

Public preprints can be crucial to early-career scientists. The delay before publication is seldom compatible with the pressure to show an impressive publication record when applying for a scholarship or a position. Increasing the perceived value of preprints as close, or equal, to journal articles will allow young researchers to put their research outcome in the open, and build a reputation for themselves through the diffusion of their work without fear that this work will not be recognized by grant or job committees.

Posting manuscripts as preprints also has the potential to improve the quality of science by allowing prepublication feedback from a large pool of reviewers. In our experience, prepublication reviews by a small network of colleagues are common in the biological sciences and form an important part of the scientific process. These “friendly” reviews increase the chance of errors being caught prior to publication. Furthermore, the formal peer-review process as a whole is critically overloaded. As the number of active scientists increases and the pressure to publish increases, it is becoming difficult for journals to find reviewers [6]. At the same time, rejection rates are high in most journals [7],[8], and when not invited to submit a revision, authors must start the process over again at another journal. As a result, initiatives to reduce time from submission to publication have emerged across the scientific community. Rohr et al. [8] called for the recycling and reuse of peer reviews: by attaching previous reviews and detailed replies to a new submission, both the editor and the referees can gauge the work done on the manuscript, and perhaps evaluate it with less prejudice. A widespread use of preprint servers can achieve the same goal of reducing the time spent in review. With a rich enough community of scientists depositing preprints, and commenting on them, the process of an open prereview can become widespread and will overall increase the quality of first submissions [9].

Finally, public preprint servers offer a fair way to establish intellectual priority by making the work available as soon as it is complete. Some manuscripts will spend much more time than others in the review process and/or in production after acceptance. This means that publication and acceptance dates do not accurately characterize who came up with an idea first. For this reason, mathematicians and physicists have embraced arXiv in part to establish priority in a fair way [2],[10].

## Preprints in Biological Sciences

**Figure 2:**
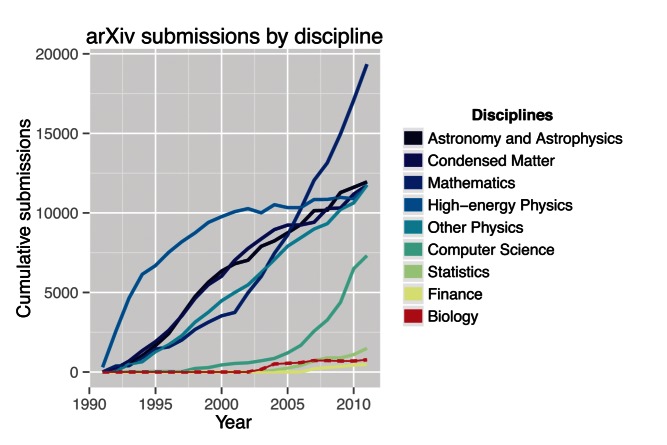
Submissions to the quantitative biology section lag behind physics, mathematics, and computer science. Data from [19].

In contrast to other disciplines, the field of biology has effectively no preprint culture, with the exception of small pockets of primarily highly quantitative research (e.g., epidemiology, population genetics). While submitting to preprint servers has become more common in the past few years, the number of biology papers submitted to preprint servers still represents only a small fraction of the total research produced in biology (Figure 2).

There are a number of reasons why biologists have not developed a culture of sharing preprints, many of which are based on common misconceptions. For example, in contrast to other fields, there is a perception in biology that public preprints make it easier to steal ideas [2]. In other fields, preprints serve the opposite role: they allow straightforward establishment of precedence, letting a researcher lay claim to an idea, thus preventing it from being “stolen” [2]. Another major concern is based on a certain interpretation of the Ingelfinger rule: scientists should not publish the same manuscript twice [11]. A preprint is simply a document that allows ideas to spread and be discussed, it is not yet formally validated by the peer-review system. This is why almost all the major publishers in biology are preprint-friendly, including: Nature Publishing Group, PLOS, BMC, PNAS, Elsevier, and Springer (Table 2). This year, both the Ecological Society of America and the Genetics Society of America changed their policies to allow public preprints. *Nature* even felt compelled to respond to the rumor that they refused manuscripts submitted to arXiv by saying that “*Nature* never wishes to stand in the way of communication between researchers. We seek rather to add value for authors and the community at large in our peer review, selection and editing” [12]. Still, a few journals adopt a “by default” hostile attitude towards preprints, mostly due to the lack of clear policy of the publishers. As an example, Wiley-Blackwell, which publishes some of the leading journals in biology, has no official policy on the matter.

**Table 2:** Policies for important publishers in biology.

Publisher	Policy
Springer	Accept
BMC	Accept
Elsevier	Accept
Nature Publishing Group	Accept
Public Library of Science	Accept
Genetics Society of America	Accept
Royal Society	Accept
National Academy of Science (USA)	Accept
Ecological Society of America	Accept
Oxford Journals	Accept
Science	Ambiguous
Wiley-Blackwell	No general policy
British Ecological Society	No answer to our query

Some publishers tolerate preprints except for a few of their medical journals, for example, the *Journal of the National Cancer Institute* from Oxford and *The Lancet* from Elsevier.

## Conclusion

The ongoing discussions on the publication process, peer review, and alternative publication models are all symptoms of the current uneasiness with the ever-growing obsession with bibliographic metrics such as the impact factor [17]. Researchers are pressured to orient their publication strategy to maximize their number of publications and total citations. A well-known consequence is to submit manuscripts first to the most prestigious journals, and then resubmit to “lower level” journals as they are rejected. The numerous negative impacts of such behavior have been discussed in depth [6] and include a long delay between the time a manuscript is finished and its publication. Research activities and the publication process are drifting away from their fundamental objective, namely the diffusion of novel scientific discoveries.

Developing a preprint culture in biology will not solve all problems with the current publication process. However, it might significantly reduce its negative consequences. The role of peer review is to judge the scientific quality of a study. It is the first barrier against the fraudulent and poor quality science that could impede scientific progress. In practice, the peer-review system is not only used to evaluate scientific quality but also to judge pertinence. On the other hand, preprints are not filtered, neither for their quality nor their pertinence. Widespread adoption of preprint servers has the potential to shift the diffusion strategy: journals would remain important to validate publications, but the relevance of a study should only be judged by many more readers than the typical two–four anonymous reviewers. With a shift in the diffusion strategy, the role of traditional journals and their editors would be to showcase scientific discoveries for specialized readership.

Making publication easier can lead to the proliferation of studies of uneven quality. A trade-off between the intensity of the peer-review filtering and the benefits to science has been hypothesized [18]. With increasingly stringent peer review, the quality of published papers can improve at the cost of an increased load on authors and reviewers and greater delays for publication. Preprints are simply bypassing this model for what we believe is the progress of science: they speed up the dissemination of scientific discoveries and put on readers' shoulders the responsibility to judge originality and pertinence.
